# Vancomycin-resistant *Enterococcus faecium* in Japan, 2007–2015: a molecular epidemiology analysis focused on examining strain characteristics over time

**DOI:** 10.1128/spectrum.02444-23

**Published:** 2023-12-15

**Authors:** Mieko Tokano, Norihito Tarumoto, Jun Sakai, Kazuo Imai, Masahiro Kodana, Toru Kawamura, Takuya Maeda, Shigefumi Maesaki

**Affiliations:** 1 Department of Infectious Disease and Infection Control, Saitama Medical University, Moroyama, Saitama, Japan; 2 Department of Allergy and Immunology, Faculty of Medicine, Saitama Medical University, Moroyama, Saitama, Japan; 3 Department of Clinical Laboratory, Saitama Medical University, Moroyama, Saitama, Japan; University of Pretoria, Pretoria, Gauteng, South Africa

**Keywords:** vancomycin-resistant enterococci (VRE), pulsed-field gel electrophoresis (PFGE), whole-genome sequencing (WGS), multilocus sequence typing (MLST), plasmid analysis, Tn1546 transposons, ST1421

## Abstract

Vancomycin-resistant enterococci (VRE) are a significant cause of healthcare-associated infections. In Japan, the estimated isolation rate of VRE is approximately 0.2%; however, sporadic outbreaks continue to occur in healthcare facilities. Despite sporadic cases of VRE at our hospital since the 2007 outbreak, it is unclear whether the same strains from 2007 were still prevalent. Therefore, we performed whole-genome sequencing of stored VRE strains at our hospital and assessed their potential utility as an alternative to pulsed-field gel electrophoresis (PFGE) for infection control. In contrast to previous studies, our analysis focused on examining strain characteristics over time. The results of taxonomic characterization using average nucleotide identity (ANI) values and multilocus sequence typing (MLST) showed good agreement with the PFGE analysis, indicating that ANI values and MLST were viable potential alternatives to PFGE. Notably, many of the strains isolated in 2010 belonged to ST1421. The first reported VRE outbreak in Japan, caused by *pstS*-null VRE ST1421, occurred in Aomori in 2018. However, the present study demonstrated, for the first time, that the ST1421 strain existed in Japan as early as 2010. In the time series analysis, the observed PFGE pattern did not correlate with the time of strain isolation, particularly for isolates collected after 2012. We also observed cases where isolates sharing the same Tn1546 variant were found on distinct clonal backgrounds. While ANI and MLST may serve as suitable alternatives to PFGE for short-term outbreak evaluations, a plasmid analysis may prove useful for long-term assessments.

**IMPORTANCE:**

Our study emphasizes the efficacy of whole-genome sequencing (WGS) in addressing outbreaks of vancomycin-resistant enterococci. WGS enables the identification and tracking of resistant bacterial strains, early detection and management of novel infectious disease outbreaks, and the appropriate selection and use of antibiotics. Furthermore, our approach deepens our understanding of how resistant bacteria transfer genes and adapt to their environments or hosts. For modern medicine, these insights have significant implications for controlling infections and effectively managing antibiotic use in the current era, where antibiotic resistance is progressing.

## INTRODUCTION

Vancomycin-resistant enterococci (VRE) were discovered in 1988 (1) and have since become a significant cause of healthcare-associated infections. Enterococci are normally present in the human body as harmless bacteria, primarily in the gastrointestinal tract (2). VRE colonization in the intestine is typically asymptomatic but can lead to a number of infections, including peritonitis, surgical wound infections, pneumonia, and sepsis, in patients with weakened immune systems. Risk factors for VRE colonization include a history of antimicrobial therapy, frequent contact with healthcare facilities, prolonged hospital stay, a weakened immune system, admission to the intensive care unit or surgical unit, and the presence of a catheter (2). Asymptomatic carriers can contribute to the spread of infections in the healthcare setting.

Recent meta-analyses have demonstrated that VRE bacteremia is associated with increased in-hospital mortality, prolonged hospital stay, and increased healthcare costs (3, 4). In addition, the mortality rate associated with VRE is 30.3% (5). Therefore, VRE infections pose a significant threat to patients receiving medical care in hospitals and long-term care facilities.

The World Health Organization has listed VRE as one of the 12 bacteria that pose the greatest threat to human health (6). Contact precautions are typically recommended as infection control measures (7), and the discontinuation of these precautions has been associated with an increase in VRE bloodstream infections (8). The transfer of vancomycin resistance can be explained by the fact that the genetic determinant for *VanA*-type vancomycin resistance resides in a mobile DNA element (e.g., Tn1546). This element can be transferred to enterococci via plasmids (9, 10). However, the significance of plasmid conjugation in nosocomial and other infections is not fully understood.

In Japan, the estimated isolation rate of VRE is approximately 0.2% (11); however, sporadic outbreaks continue to occur in healthcare facilities. Multilocus sequence typing (MLST) of *Enterococcus faecium* relies on sequencing seven essential housekeeping genes (12). However, there are strains of *E. faecium* that do not harbor the *pstS* housekeeping gene in the MLST allele [e.g., sequence type (ST) 1421 and ST1424] (13). Although these *pstS*-null sequence types have emerged globally in recent years, there have been limited reports in Japan (14).

Although sporadic cases of VRE have persisted at our hospital since the 2007 outbreak, whether the same strain was present as in 2007 remains unclear. Therefore, we conducted whole-genome sequencing (WGS) of the stored VRE strains at our hospital and conducted a WGS analysis as a substitute for pulsed-field gel electrophoresis (PFGE) for infection control.

## MATERIALS AND METHODS

### Patients and bacterial isolates

Patients with VRE detected in sputum, urine, stool, and rectal swab samples who were admitted to Saitama Medical University Hospital, a 1,000-bed emergency hospital in Saitama, Japan, between 2007 and 2015 were included in this study. If more than one case was detected in the same ward within 4 weeks, only the first case was included. Patient specimens were incubated in a VRE-selective medium (Becton Dickinson, Franklin Lakes, NJ, USA) for 48 h. All isolates derived from cultures were identified by a MALDI Biotyper with the MALDI Biotyper 3.1 software program and MALDI Biotyper Reference Library version 4.0.0.1 (Bruker Daltonics, Bremen, Germany) according to the manufacturer’s instructions in an autoflex speed mass spectrometer (Bruker Daltonics), and all isolates were kept frozen at −80°C until use. Antimicrobial susceptibility testing was performed using a MicroScan WalkAway 96 Plus (Beckman Coulter, Brea, CA, USA). Information on baseline demographic and clinical characteristics, treatments administered, and clinical outcomes was obtained from electronic medical records.

The study was approved by the Institutional Review Board of Saitama Medical University Hospital and was conducted in accordance with the tenets of the Declaration of Helsinki. The requirement for informed consent was waived because this was a retrospective study.

### PFGE analyses

Frozen VRE strains were incubated in the BHI medium for 3.5 h for bacterial recovery. *SmaI*-PFGE was performed as previously described (15, 16). Electrophoresis was performed using CHEF-Mapper (Bio-Rad, Hercules, CA, USA). A cluster analysis was performed using the GelCompar II software program (Applied Maths, Sint-Martens-Latem, Belgium) for band matching with 0.87% position tolerance. A similarity analysis was performed using the Jaccard coefficient, and a cluster analysis was performed using the unweighted pair group method with arithmetic averages.

### Whole-genome sequencing

After overnight culture, genomic DNA was extracted using the Illumina Nextera XT Library Prep Kit (Illumina, San Diego, CA, USA) according to the manufacturer’s protocol and run on a MiSeq sequencer (Illumina) to generate 250 bp paired-end reads. We used FastQC for quality control (17). The reads were trimmed using Trimmomatic version 0.39 (5), and bbmap (version 35.34) was used to calculate the average coverage (18). We analyzed the depth of coverage of more than 50× high-quality genomes. *De novo* sequencing was performed using the Spades software program (version 13) (19). MLST was determined from *de novo* assemblies using the MLST software program from the Center for Genomic Epidemiology website (20). Clustering was performed using the Gegenees software program (21). A phylogenetic tree analysis was performed using the CSI Phylogeny 1.4 software program (22). Tn1546 transposon structures carrying *vanA* were identified by comparison with the reference sequence of Tn1546 (GenBank M97297.1). Genome mapping was performed using the BWA software program (23).

## RESULTS

### Clinical characteristics

Twenty-nine patients were enrolled in the study. The patient characteristics are shown in Table 1. Thirteen patients (44.8%) were transferred from other hospitals, including five from the same hospital. Twenty-four patients (82.8%) had previous hospitalization within the previous year. Nine patients (31.0%) received ≥2 antibiotics. Three of these patients received vancomycin for short periods of time (≤2 weeks).

**TABLE 1:** Baseline characteristics of the 29 patients

Characteristics of patients	Total = 29	
	*n*	%
Age, median (years)	69	
Male sex	14	48.3
Comorbidity dialysis		
Diabetes mellitus	8	27.6
Malignancy	11	37.9
End-stage renal disease	13	44.8
Gastrointestinal disease	10	34.5
Other chronic diseases	28	96.6
Transfer from a convalescent hospital	13	44.8
Receipt of ≥2 different classes of antibiotics within 30 days	9	31.0
Episodes of hospitalization within 1 year	24	82.8
Age > 60 years	23	79.3
Immunosuppressive	8	27.6
Admission to the intensive care unit	2	6.90
Vancomycin-resistant enterococci isolated from cultures of clinical specimens other than stool or rectal swab	4	13.8

### Analyses of VRE strains

**Fig 1:**
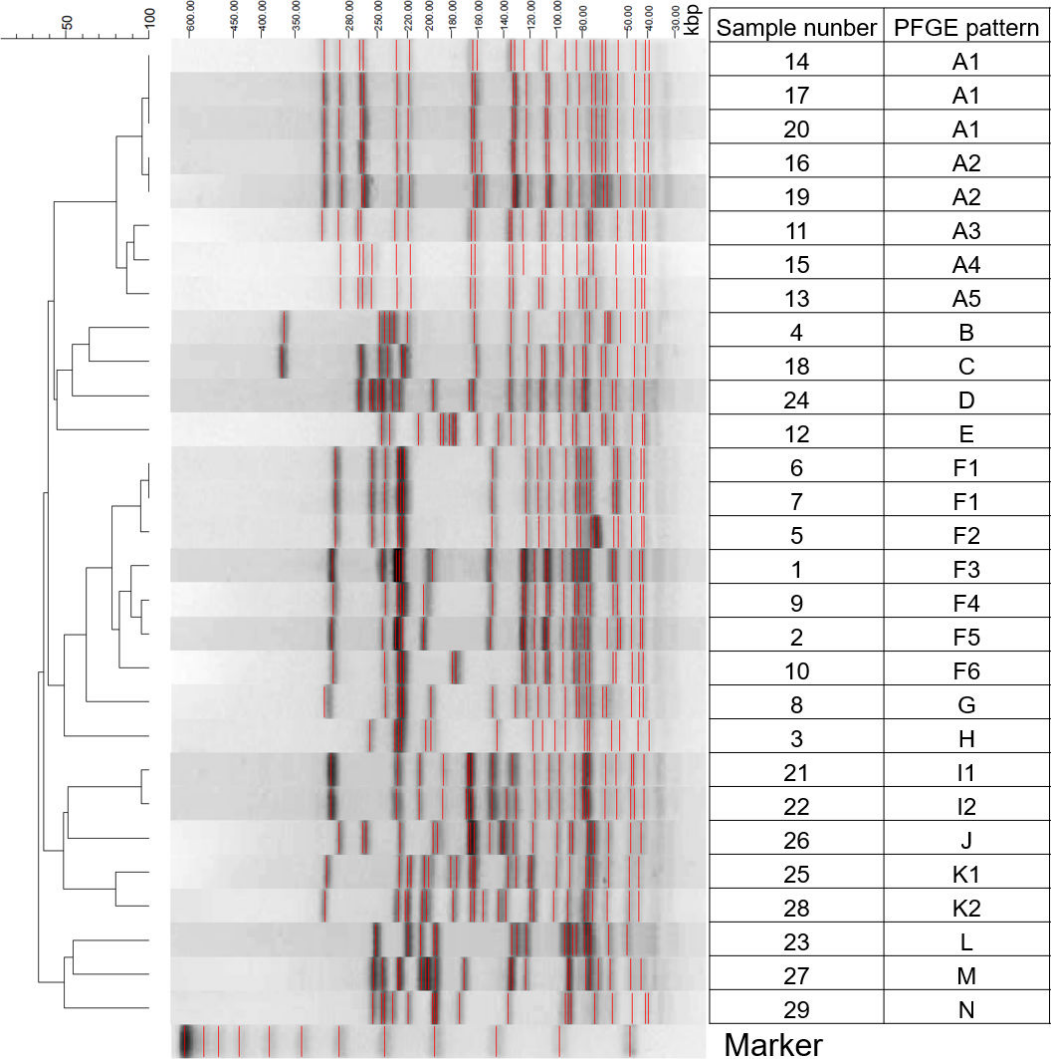
PFGE profiles obtained by *SmaI* digestion of 29 isolated VRE strains. kbp: kilobase pairs; marker: DNA size marker.

For the microbial ecological analysis, 29 strains of VRE were subjected to a clustering analysis based on a PFGE pattern analysis (Fig. 1). Raw sequence data were assembled into contigs. Seven housekeeping loci were selected for the characterization of *E. faecium* isolates by MLST (12). The PFGE pattern, MLST results, Tn1546 characterization, and sampling year are shown on the right-hand side of Table 2. A total of 15 of the 29 VRE isolates were assigned to one of the two PFGE groups. The remaining isolates exhibited unique PFGE patterns. In the time series analysis, no PFGE pattern characteristics were observed, especially with shares separated after 2012.

**TABLE 2:** The analysis of VRE strains and Tn1546 characterization

Year of sampling	Sample no.	PFGE pattern	MLST	Tn1546 characterization											
				Tn1546 type	IRL	Transposase	Resolvase	vanR	vanS	vanH	vanA	vanX	vanY	vanZ	IRR
2007	1^*c*^	F3^*d*^	78	Ab	−	1459–2967	＋	＋	＋	＋	＋	＋	＋	−	−
2007	2^*c*^	F5^*d*^	78	Ab	−	1459–2967	＋	＋	＋	＋	＋	＋	＋	−	−
2007	3	H	78	Ac	−	1390–2967	＋	＋	＋	＋	＋	＋	＋	−	−
2008	5^*c*^	F2^*d*^	78	Ad	−	1507–2967	＋	＋	＋	＋	＋	＋	1–783	−	−
2008	6^*c*^	F1^*d*^	78	Aa	−	1507–2967	＋	＋	＋	＋	＋	＋	＋	−	−
2008	7^*c*^	F1^*d*^	78	Ac	−	1390–2967	＋	＋	＋	＋	＋	＋	＋	−	−
2008	8^*c*^	G^*d*^	78	Ac	−	1390–2967	＋	＋	＋	＋	＋	＋	＋	−	−
2009	9^*c*^	F4^*d*^	78	Ab	−	1459–2967	＋	＋	＋	＋	＋	＋	＋	−	−
2009	10^*c*^	F6^*d*^	78	Ab	−	1459–2967	＋	＋	＋	＋	＋	＋	＋	−	−
2010	4	B	17^*a*^	Ac	−	1390–2967	＋	＋	＋	＋	＋	＋	＋	−	−
2010	11^*b*^	A3^*e*^	1421	B	−	−	−	＋	＋	＋	＋	＋	1–901	−	−
2010	12	E	323	B	−	−	−	＋	＋	＋	＋	＋	1–901	−	−
2010	13	A5^*e*^	1421	B	−	−	−	＋	＋	＋	＋	＋	1–901	−	−
2010	14^*b*^	A1^*e*^	1421	B	−	−	−	＋	＋	＋	＋	＋	1–901	−	−
2010	15^*b*^	A4^*e*^	1421	B	−	−	−	＋	＋	＋	＋	＋	1–901	−	−
2010	16^*b*^	A2^*e*^	1421	B	−	−	−	＋	＋	＋	＋	＋	1–901	−	−
2010	17^*b*^	A1^*e*^	1421	B	−	−	−	＋	＋	＋	＋	＋	1–901	−	−
2010	18	C	17	B	−	−	−	＋	＋	＋	＋	＋	1–901	−	−
2010	19^*b*^	A2^*e*^	1421	B	−	−	−	＋	＋	＋	＋	＋	1–901	−	−
2010	20^*b*^	A1^*e*^	1421	B	−	−	−	＋	＋	＋	＋	＋	1–901	−	−
2012	21	I1	17	B	−	−	−	＋	＋	＋	＋	＋	1–901	−	−
2013	22	I2	78	B	−	−	−	＋	＋	＋	＋	＋	1–901	−	−
2012	23	L	18	B	−	−	−	＋	＋	＋	＋	＋	1–901	−	−
2013	24	D	17	B	−	−	−	＋	＋	＋	＋	＋	1–901	−	−
2013	25	K1	78	B	−	−	−	＋	＋	＋	＋	＋	1–901	−	−
2013	26	J	78	B	−	−	−	＋	＋	＋	＋	＋	1–901	−	−
2014	27	M	17	C	−	−	−	-	＋	＋	＋	＋	1–901	−	−
2014	28	K2	78	B	−	−	−	＋	＋	＋	＋	＋	1–901	−	−
2015	29	N	612	D	−	＋	＋	＋	＋	＋	＋	＋	＋	＋	−

^*a*^ This strain is a subspecies of ST17.

^*b*^ Cluster 2.

^*c*^ Cluster 1.

^*d*^ PFGE group 1.

^*e*^ PFGE group 2.

The MLST analysis revealed that almost all the VRE isolates could be assigned to known MLST types. Sample 4 contained a single-nucleotide polymorphism (SNP) in the *gdh* gene. This strain is a subspecies of ST17. The predominant STs were ST78 (*n* = 13, 44.8%), ST1421 (*n* = 8, 27.6%), and ST17 (*n* = 5, 17.2%). These sequence types belong to clonal complex 17. PFGE groups 1 and 2 correspond to ST1421 and ST78, respectively.

**Fig 2:**
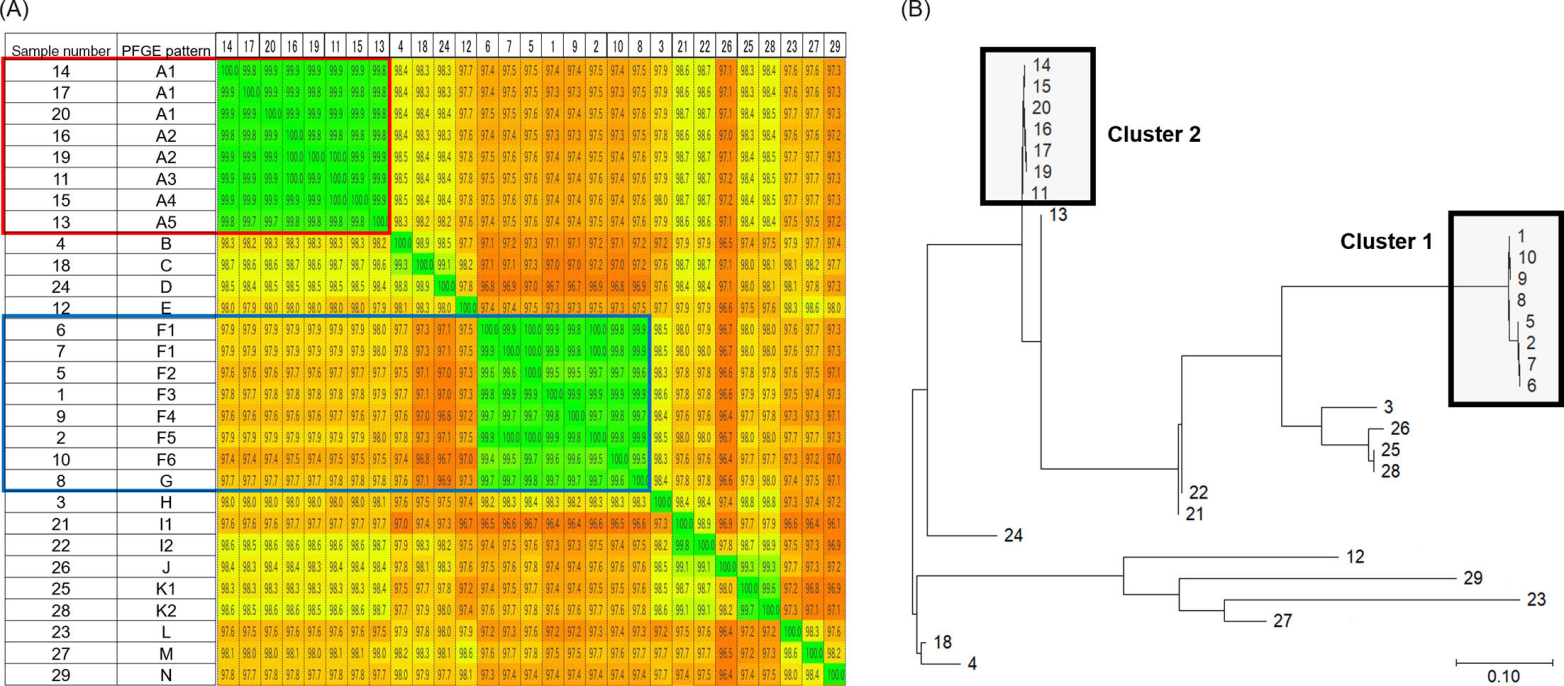
(**A**) Whole-genome ANI values of the 29 strains. (**B**) Core genome SNP phylogenetic tree of 29 isolates. Sample numbers are the same as listed in Fig. 1; Table 2.

Homology was evaluated using raw sequence data. Taxonomic characterization through similarity comparisons using ANI values estimated for the 29 strains of VRE is shown in Fig. 2A. These results are in good agreement with the PFGE analysis. The pairwise ANI values of PFGE groups 1 and 2 were >99.8% and >99.4%, respectively, suggesting that these strains were subspecies.

The phylogenetic tree based on the core genome single-nucleotide variants of the same 29 isolates is shown in Fig. 2B. This result is generally in agreement with the PFGE analysis, and two clusters were formed.

### Tn1546 characterization

Next, mapping of the Tn1546 elements revealed seven different transposon types (Table 2, left). The numbers indicate that the sample contained only part of the sequence. These results are not in good agreement with the PFGE analysis. All the VRE isolates carried the *VanS*, *VanH*, *VanA*, and *VanX* genes. In the time series analysis, type A was predominant until 2009, whereas type B was the most predominant from 2010 onward.

## DISCUSSION

We experienced an outbreak in 2007 that was triggered by the detection of VRE in one patient’s catheter tip culture and another patient’s fecal surveillance culture. Additionally, we experienced a second outbreak in 2010. Asymptomatic carriers may shed VRE for a long period (24). Therefore, we continued screening tests even after the outbreak had been contained. Indeed, in our hospital, VRE was detected sporadically, even after the outbreak ended. We need to use appropriate methods for bacterial genotyping to control infection. In the present study, we analyzed VRE isolates collected between 2007 and 2015 using PFGE, WGS, and MLST. Many previous studies have analyzed only transient clusters that occur in specific wards or facilities. Unlike previous studies, this study analyzed strain characteristics over time. In addition, this study included strains obtained from outbreaks as well as strains from screening tests at admission.

The results of taxonomic characterization through similarity comparisons using ANI values and MLST were in good agreement with the PFGE analysis. WGS directly determines the nucleotide sequence of the DNA sample, providing extremely high resolution and accuracy. In addition, WGS can be used to detect and identify mutations and genetic abnormalities by sequencing the entire genome. A previous study suggested that WGS-ANI has increased discriminatory power in comparison to MLST and PFGE and better epidemiological concordance than PFGE (25). Accordingly, ANI and MLST can be substituted for PFGE in these evaluations. PFGE group 1 corresponded to ST1421. This clone lacked the *pstS* housekeeping gene of the MLST allele. *pstS*-null sequence types were first reported in Australia in 2016 (26). The first VRE outbreak in Japan due to *pstS*-null VRE ST1421 was reported in Aomori in 2018 (14). The ST1421 strain was rarely detected in Japan but was detected in our 2010 cluster. We were able to prove, for the first time, that the ST1421 strain already existed in Japan as of 2010. In fact, *pstS*-null sequence types had already appeared in Korea in 2006 (27). We suspect that the ST1421 strain may have entered Japan via foreign countries before 2010 and that it was not detected in the tests. We must be vigilant regarding the importation of resistant bacteria by travelers.

In the time series analysis, the PFGE pattern did not coincide with the time of isolation of the strains, especially with the shares separated after 2012. We observed that cases of isolates shared an identical Tn1546 variant on distinct clonal backgrounds. Time series analyses of Tn1546 characterization showed that type A was predominant until 2009, whereas type B was the most predominant from 2010 onward. It is likely that the 2010 outbreak was a separate episode from the 2007 outbreak. The nine isolates collected after 2012 did not belong to a specific PFGE pattern group or a specific cluster. Therefore, these isolates can be attributed to different environments. It is also likely that Tn1546 (or a plasmid containing Tn1546) may have been transferred between the strains for strains isolated after 2010. This plasmid may have been prevalent in the area where our hospital exists. Plasmid analyses assist in tracking the spread of plasmids among different microbial species and monitoring how microorganisms adapt to different environments or hosts. Therefore, it is necessary to combine WGS with plasmid analyses.

The present study was associated with several limitations. First, the study was performed at a single facility, which may have introduced a selection basis. Second, plasmid assembly and genotyping were not performed. In previous studies, plasmid typing was conducted, but the mapping of Tn1546 in the present study also provided some information. PFGE remains the gold standard for molecular epidemiological investigations of nosocomial outbreaks of VRE (25). However, plasmid analyses may have allowed us to evaluate not only nosocomial but also regional epidemics. Therefore, a plasmid analysis may be superior to PFGE. Although ANI and MLST may be useful substitutes for PFGE for short-term outbreak evaluations, plasmid analyses may be more useful for long-term evaluations.

## Data Availability

The genome sequence was deposited in DDBJ(SRA) under the accession number DRA017150.
